# Evaluating the effects of control interventions and estimating the inapparent infections for dengue outbreak in Hangzhou, China

**DOI:** 10.1371/journal.pone.0220391

**Published:** 2019-08-08

**Authors:** Haocheng Wu, Chen Wu, Qinbao Lu, Zheyuan Ding, Ming Xue, Junfen Lin

**Affiliations:** 1 Zhejiang Province Center for Disease Control and Prevention, Hangzhou, Zhejiang Province, China; 2 Key Laboratory for Vaccine, Prevention and Control of Infectious Disease of Zhejiang Province, Hangzhou, Zhejiang Province, China; 3 Hangzhou Centre for Disease Control and Prevention, Hangzhou, Zhejiang, Province, China; Institut Pasteur, FRANCE

## Abstract

**Background:**

The number of dengue fever (DF) cases and the number of dengue outbreaks have increased in recent years in Zhejiang Province, China. An unexpected dengue outbreak was reported in Hangzhou in 2017 and caused more than one thousand dengue cases. This study was undertaken to evaluate the effectiveness of the actual control measures, estimate the proportion of inapparent infections during this outbreak and simulate epidemic development based on different levels of control measures taking inapparent infections into consideration.

**Methods:**

The epidemic data of dengue cases in Hangzhou, Zhejiang Province, in 2017 and the number of the people exposed to the outbreaks were obtained from the China Information Network System of Disease Prevention and Control. The epidemic without intervention measures was used to estimate the unknown parameters. A susceptible-exposed-infectious/inapparent-recovered (SEIAR) model was used to estimate the effectiveness of the control interventions. The inapparent infections were also evaluated at the same time.

**Results:**

In total, 1137 indigenous dengue cases were reported in Hangzhou in 2017. The number of indigenous dengue cases was estimated by the SEIAR model. This number was predicted to reach 6090 by the end of November 2, 2017, if no control measures were implemented. The total number of reported cases was reduced by 81.33% in contrast to the estimated incidence without intervention. The number of average daily inapparent cases was 10.18 times higher than the number of symptomatic cases. The earlier and more rigorously the vector control interventions were implemented, the more effective they were. The results showed that implementing vector control continuously for more than twenty days was more effective than every few days of implementation. Case isolation is not sufficient enough for epidemic control and only reduced the incidence by 38.10% in contrast to the estimated incidence without intervention, even if case isolation began seven days after the onset of the first case.

**Conclusions:**

The practical control interventions in the outbreaks that occurred in Hangzhou City were highly effective. The proportion of inapparent infections was large, and it played an important role in transmitting the disease during this epidemic. Early, continuous and high efficacy vector control interventions are necessary to limit the development of a dengue epidemic. Timely diagnosis and case reporting are important in the intervention at an early stage of the epidemic.

## Introduction

Dengue is an arthropod-borne viral disease that results in a broad spectrum of clinical symptoms ranging from mild fever to dengue hemorrhagic fever (DHF), which can lead to dengue shock syndrome (DSS) and death [1]. Dengue has become a major public health problem since its incidence has increased >30-fold in recent decades [2,3]. Recent estimates of dengue disease burden have indicated that there are 390 million dengue infections and 96 million symptomatic infections in the world per year, of which 2 million are severe cases and 21,000 result in death[3–5]. Furthermore, the direct and indirect costs resulting from this disease have reached 8.9 billion US dollars annually [6]. Despite being considered an imported disease in mainland China, dengue fever (DF) cases and the number of dengue outbreaks have increased in recent years [7,8]. Located in the Yangtze River Delta region of southeast China, Zhejiang has a typical subtropical climate, which is favorable for the growth of *Aedes* mosquitoes [8]. There have been a number of DF outbreaks in Zhejiang since 2004 (83 cases in Cixi County, 196 cases in Yiwu County and 1138 cases in Hangzhou)[8–10]. During those outbreaks, control measures were always implemented, including enhancing leadership, conducting risk assessment, monitoring adult mosquito density and the Breteau Index, eradicating the vector, and accelerating the process of dengue case diagnosis, reporting and treatment [11]. However, it is unknown whether these intensified control measures were effective and how they affected the development of dengue epidemics.

To fill this gap in research, mathematical models were employed to test the effectiveness of the control measures used in DF outbreaks in Zhejiang. Mathematical models are helpful in improving understanding of transmission dynamics, vector behavior and effectiveness of control strategies to combat DF [2]. In particular, the compartment model has been applied widely to assess the effectiveness of control measures for many infectious diseases, including chikungunya fever[12], Ebola[13], hemorrhagic conjunctivitis[14], shigellosis[15], influenza[16] and norovirus[17].

For dengue, researchers have used transmission models to examine the role of climate in driving vector dynamics and dengue virus transmission [18–20]. Some research has focused on the dynamics of vertical and mechanical transmission [21,22]. Transmission models have also been used to develop a stochastic spatial model[23,24], to evaluate control strategies[11,25–26] and to simulate agent-based models[27].

However, research has not considered incorporating dengue with inapparent infections in a transmission model, which might greatly influence dengue transmission in outbreaks [28]. According to research, dengue infections with no detectable symptoms or before the onset of symptoms are significantly more infectious to mosquitoes than people with symptomatic infections. The inapparent cases could contribute to the persistent circulation of dengue virus and may play an important role in driving epidemics such as cholera[29]. Therefore, it is necessary to consider the role of inapparent infections in dengue epidemics. The objective of this study was threefold. First, this study evaluated the effectiveness of vector control and case isolation. Second, it simulated epidemic development based on different levels of control measures, taking inapparent infections into consideration. Lastly, it estimated the proportion of inapparent infections.

## Materials and methods

### Ethical review

This study was reviewed and approved by the Ethics Committee of the Zhejiang Provincial Center for Disease Control and Prevention. All the data of the individuals were kept confidential as requested. Verbal informed consent was obtained from all patients before diagnosis and reporting to the China Information Network System of Disease Prevention and Control. All the methods used in the study were in accordance with the applicable guidelines and regulations.

### Profile of Hangzhou City

Hangzhou City is located in southeast China between longitudes 118^o^E-120^o^E and latitudes 29^o^N-30^o^N. There are 13 counties in Hangzhou: Shangcheng, Xiacheng, Gongshu, Jianggan, Xihu, Yuhang, Binjiang, Xiaoshan, Fuyang, Linan, Tonglu, Jiande and Chunan.

### Data collection

Human dengue cases diagnosed in the hospital must be reported to the China Information Network System of Disease Prevention and Control by the medical staff. The epidemic data of the dengue cases in Hangzhou, Zhejiang, in 2017 and the number of the people exposed to the outbreaks were obtained from this network system. Individual information on cases and deaths was imported. Surveillance information, including demographic characteristics and geographic and temporal distributions, was computed by the system. The definition of reporting cases referred to the ‘Diagnostic criteria for dengue fever’ (WS 216–2008) of China.

### The epidemiological features of outbreaks in Hangzhou

**Fig 1:**
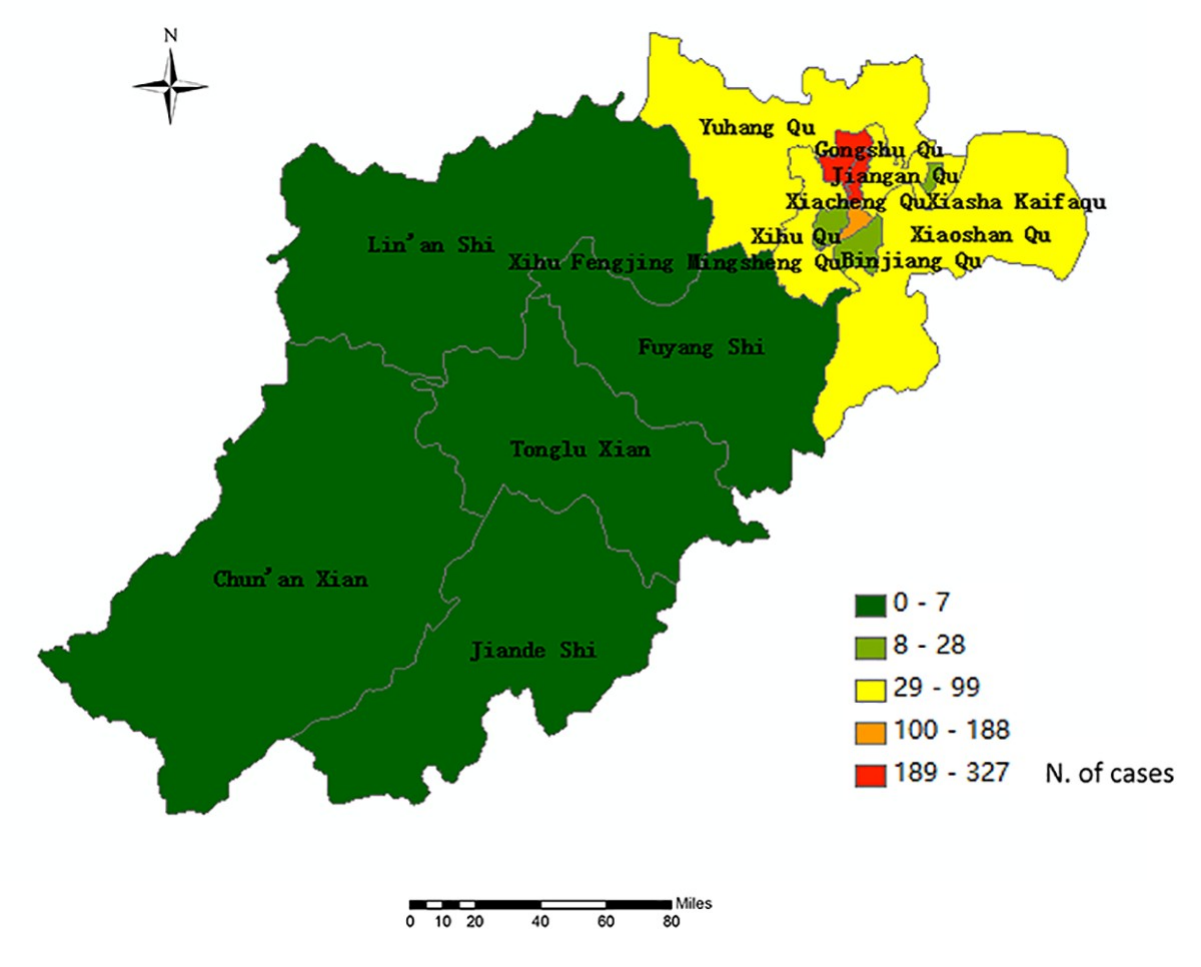
Map of the incidence of dengue at the county level in Hangzhou City, China, 2017.

A dengue outbreak occurred Hangzhou, Zhejiang Province in 2017. There were 1137 indigenous dengue cases reported in Hangzhou, 951 of which were confirmed cases and 186 clinical cases. The first case began on July 15, and the last case was onset on November 2, 2017. However, the index case was only diagnosed and reported on August 22. The number of cases increased rapidly from August 20, reached a peak between August 26 and September 11, and then declined to less than 10 cases per day within two weeks (Fig 1). The proportion of gender was 1.01:1, with 572 male cases and 565 female cases reported. The median age was 51. Nearly 52% of the patients were more than 50 years old. Thirty-nine percent of the patients were workers (Fig 1).

### Spatial autocorrelation analysis

A global autocorrelation analysis using Moran’s I was carried out to identify spatial autocorrelations. According to the theory, the closer two locations were to each other, the more likely the incidence rates would have an impact on each other [30]. The global autocorrelation demonstrates the spatial distribution clusters over areas [31].

The semivariance parameter was also used to evaluate the strength of the correlation, except for Moran's I Index. The parameters of the semivariance function and the nugget effect can be estimated by an empirical semivariance function[32]:

$$\gamma (h)=\frac{1}{2N(h)}\sum_{i=1}^{N(h)}{\left[Z_{obs}(x_{i}+h_{x},y_{i}+h_{y})‐Z_{obs}(x_{i},y_{i})\right]}^{2}$$ (1)

where γ(h) is the semivariance value at the distance interval h, N(h) is the number of sample pairs within the distance interval h, and Z_obs_(x_i_+h_x_+y_i_+h_y_) Z_obs_(x_i_,y_i_) are sample values at two points separated by the distance interval h.

### The basic model

Dengue virus is maintained in an endemic–epidemic cycle involving human and mosquitoes and is fully adapted to humans [3]. The female mosquitoes become infected when they bite a symptomatic case or an inapparent infection. After the extrinsic incubation period, once the salivary gland is infected, the mosquitoes remain infective for life and can transmit the virus to individuals they subsequently probe. Since the epidemiological importance of vertical transmission is uncertain, we did not add this pattern of transmission into our model [3]. Furthermore, we did not take the change in population into consideration, given that the outbreak that occurred in Hangzhou only lasted 3 months. Because inapparent cases may play an important role in driving epidemics, we incorporated inapparent cases into the susceptible-exposed-infectious-recovered (SEIR) model. Hence, a susceptible-exposed-infectious/inapparent-recovered (SEIAR) model [11,12,16,33–35] was used to simulate the outbreak epidemic. The mathematical formula of the model is illustrated as follows:

$$\frac{dS_{h}}{dt}=-\beta_{vh}\frac{I_{V}}{N_{h}}S_{h}$$ (2)

$$\frac{dE_{h}}{dt}=\beta_{vh}\frac{I_{V}}{N_{h}}S_{h}-\alpha_{h1}pE_{h}-\alpha_{h2}(1-p)E_{h}$$ (3)

$$\frac{dI_{h}}{dt}=\alpha_{h1}pE_{h}-\gamma_{h1}I$$ (4)

$$\frac{dA_{h}}{dt}=\alpha_{h2}(1-p)E_{h}-\gamma_{h2}A$$ (5)

$$\frac{dR_{h}}{dt}=\gamma_{h1}I+\gamma_{h2}A$$ (6)

$$\frac{dS_{v}}{dt}=r_{v}(1-\frac{N_{v}}{K})N_{v}-\beta_{hv}\frac{(1-Q)I_{h}+A_{h}}{N_{h}}S_{v}-(\mu_{v}+\delta )S_{v}$$ (7)

$$\frac{dE_{v}}{dt}=\beta_{hv}\frac{(1-Q)I_{h}+A_{h}}{N_{h}}S_{v}-(\alpha_{V}+\mu_{v}+\delta )S_{v}$$ (8)

$$\frac{dI_{v}}{dt}=\alpha_{v}E_{v}-(\mu_{v}+\delta )I_{v}$$ (9)

The variables for the Eqs (1–8) are shown in Table 1. *dS*_*h*_/*dt*, *dE*_*h*_/*dt*, *dI*_*h*_/*dt*, *dA*_*h*_/*dt*, *dR*_*h*_/*dt*, *dS*_*v*_/*dt*, *dE*_*v*_/*dt* and *dI*_*v*_/*dt* refer to changing rates of the *S*_*h*_, *E*_*h*_, *I*_*h*_, *A*_*h*_, *R*_*h*_, *S*_*v*_, *E*_*v*_ and *I*_*v*_ at time t, respectively.

**Table 1:** Variables for the SEIAR equation models.

Variables	Description	Unit	Value	Source
*S*_*h*_	Number of susceptible humans	*person*	-	-
*E*_*h*_	Number of exposed humans	*person*	-	-
*I*_*h*_	Number of symptomatic humans	*person*	-	-
*A*_*h*_	Number of inapparent humans	*person*	-	-
*R*_*h*_	Number of recovered humans	*person*	-	-
*S*_*v*_	Number of susceptible mosquitoes	*mosquito*	-	-
*E*_*v*_	Number of exposed mosquitoes	*mosquito*	-	-
*I*_*v*_	Number of infectious mosquitoes	*mosquito*	-	-
*N*_*h*_	Total human population size	*person*	611209	Reporting
*N*_*v*_	Total mosquito population size	*mosquito*	-	-
*β*_*hv*_	Transmission rate from an infected human to a susceptible mosquito	1	0.312	Curve fitting
*β*_*vh*_	Transmission rate from an infected mosquito to a susceptible human	1	0.584	Curve fitting
*α*_*h*1_	Changing rate of humans from the exposed state to the symptomatic state	*day*^−1^	1/5	References[11,23]
*α*_*h*2_	Changing rate of humans from the exposed state to the inapparent state with infectious ability	*day*^−1^	0.377	Curve fitting
*α*_*v*_	Changing rate of mosquitoes from the exposed state to the infectious state	*day*^−1^	1/10	References[11,23]
*γ*_*h*1_	Recovery rate of human from the symptomatic state to the recovered state	*day*^−1^	1/6	References[11,23]
*γ*_*h*2_	Changing rate of human from the inapparent state with infectious ability to the state with no infectious ability	*day*^−1^	0.274	Curve fitting
*μ*_*v*_	Density-independent death rate for mosquitoes	*day*^−1^	1/21	References[11,23]
*p*	Proportion of exposed humans to symptomatic humans	1	0.156	Curve fitting
*δ*	Reduction rate of mosquitoes due to vector control measures	*day*^−1^	-	Setting
*K*	Carrying capacity of mosquitoes	*mosquito*	805355	Curve fitting
*Q*	Proportion of isolation	1	100%	Setting
*r*_*v*_	Natural growth rate of mosquitoes	*day*^−1^	0.002	Curve fitting
*S*_*v*0_	Initial values of susceptible mosquitoes	*mosquito*	3489860	Curve fitting
*E*_*v*0_	Initial values of exposed mosquitoes	*mosquito*	1	Curve fitting
*I*_*v*0_	Initial values of infectious mosquitoes	*mosquito*	1	Curve fitting

*h*: human; *v*: mosquitoes; *hv*: transmission from human to mosquitoes; *vh*: transmission from mosquitoes to human.

### Estimation of parameters

The total human population was reported from the system. Ten variables, including *β*_*hv*_, *β*_*vh*_, *α*_*h*2_, *γ*_*h*2_, *p*, *K*, *r*_*v*_, *S*_*v*0_, *E*_*v*0_ and *I*_*v*0_, would be unknown and unstable from different outbreaks. Therefore, these ten variables were estimated and optimized from the model. The method used was the Runge-Kutta method of order 4. The other four variables, including *α*_*h*1_, *α*_*v*_, *γ*_*h*1_, and *μ*_*v*_, were generally known and stable. Thus, they were referred to related studies[11,36]. All the parameters are shown in Table 1. The reduction rate of mosquitoes as a result of the vector control measures (*δ*) was set to 0, -log0.95, -log0.925 and -log0.90, which means without vector control measures, 5% daily reduction in mosquito density at day t, 7.5% daily reduction in mosquito density at day t and 10% daily reduction in mosquito density at day t, respectively. The proportion of isolation (Q) was set to 100%, which means every symptomatic case should be isolated in time, while 0 means no isolation (Table 1).

### Simulation steps

The outbreaks that occurred in Hangzhou were divided into two periods with the cut-off set as August 29. The period before August 29 was regarded as without intervention measures, and the integrated measures were implemented after the date. First, we simulated the epidemic without any control measures based on the data of the first stage and obtained the unknown parameters. Then, the mean absolute percentage error (MAPE) was calculated to check the accuracy of the model by comparing the reported number of cases with the estimated incidence among the first period. Third, we simulated a model without any control measures to estimate the total possible cases and the inapparent incidence, and the inapparent-to-symptomatic (I:S) ratio was computed at the same time. Fourth, we estimated the effectiveness of vector control with different levels of daily reduction in mosquito density and at different initial times. Due to the limited control resources, the epidemic with implementing control measures between an interval period was also simulated. Finally, the effectiveness of case isolation was evaluated at different initial times.

### Sensitivity analysis

The four parameters of the SEIAR model, *α*_*h*1_, *α*_*v*_, *γ*_*h*1_, *μ*_*v*_, were adapted from the literature. Uncertainty may exist for the simulation. Therefore, sensitivity analysis was performed by changing four parameters. The theoretical range of each parameter was split into 1,000 values based on the epidemiological characteristics of dengue. The changing rate of humans from the exposed state to the symptomatic state, the changing rate of mosquitoes from the exposed state to the infectious state, the recovery rate of humans from the symptomatic state to the recovered state and the density-independent death rate for mosquitoes were 0.14–0.25 (4–7 days), 0.07–0.14 (7–14 days), 0.08–0.25 (4–12 days) and 0.02–0.13 (8–42 days), respectively[36].

### Statistical analysis

ArcGIS software (version 10.1, SERI Inc.; Redlands, CA, USA) was used for the spatial autocorrelation analysis. County was adopted as the geographic unit to calculate the spatial autocorrelation. The Runge-Kutta method of order 4 with the tolerance set at 0.001 was used to perform curve fitting. These methods were computed by Berkeley Madonna 8.3.18 (University of California at Berkeley, Berkeley, USA). The best fitting parameters were computed automatically by this software [33–35,37].

## Result

### Spatial autocorrelation

**Fig 2:**
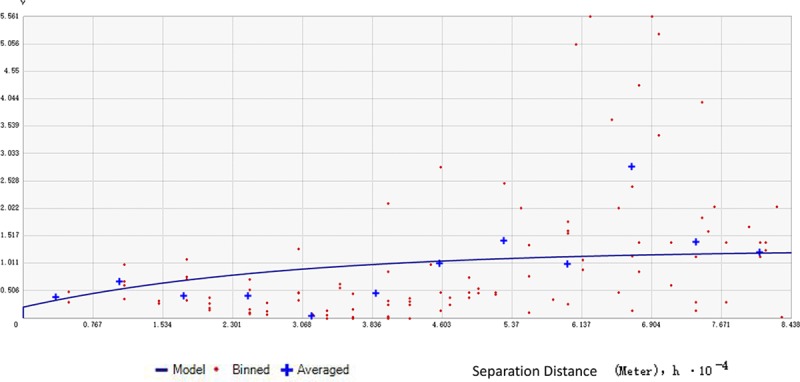
The semivariogram of dengue outbreak in Hangzhou City, 2017.

According to the global autocorrelation analysis, the distribution of the dengue cases in the outbreak presented a spatial autocorrelation. The Moran's I Index was 0.2097 (*Z* score = 2.7366, *P* value<0.001). In addition, it was shown in the semivariogram figure that the value of the semivariation function was relatively low over a short range. The value gradually increased as the distance between the sample points grew and fluctuated around an extremum when the distance grew to a particular value. The partial sill (C_1_) was 1.053. The nugget (C_0_) was 0.2055. The C_1_/ C_0_+ C_1_ ratio was 83.67%. This ratio suggested that the spatial autocorrelation was relatively strong in general (Fig 2).

### A simulated epidemic without intervention

**Fig 3:**
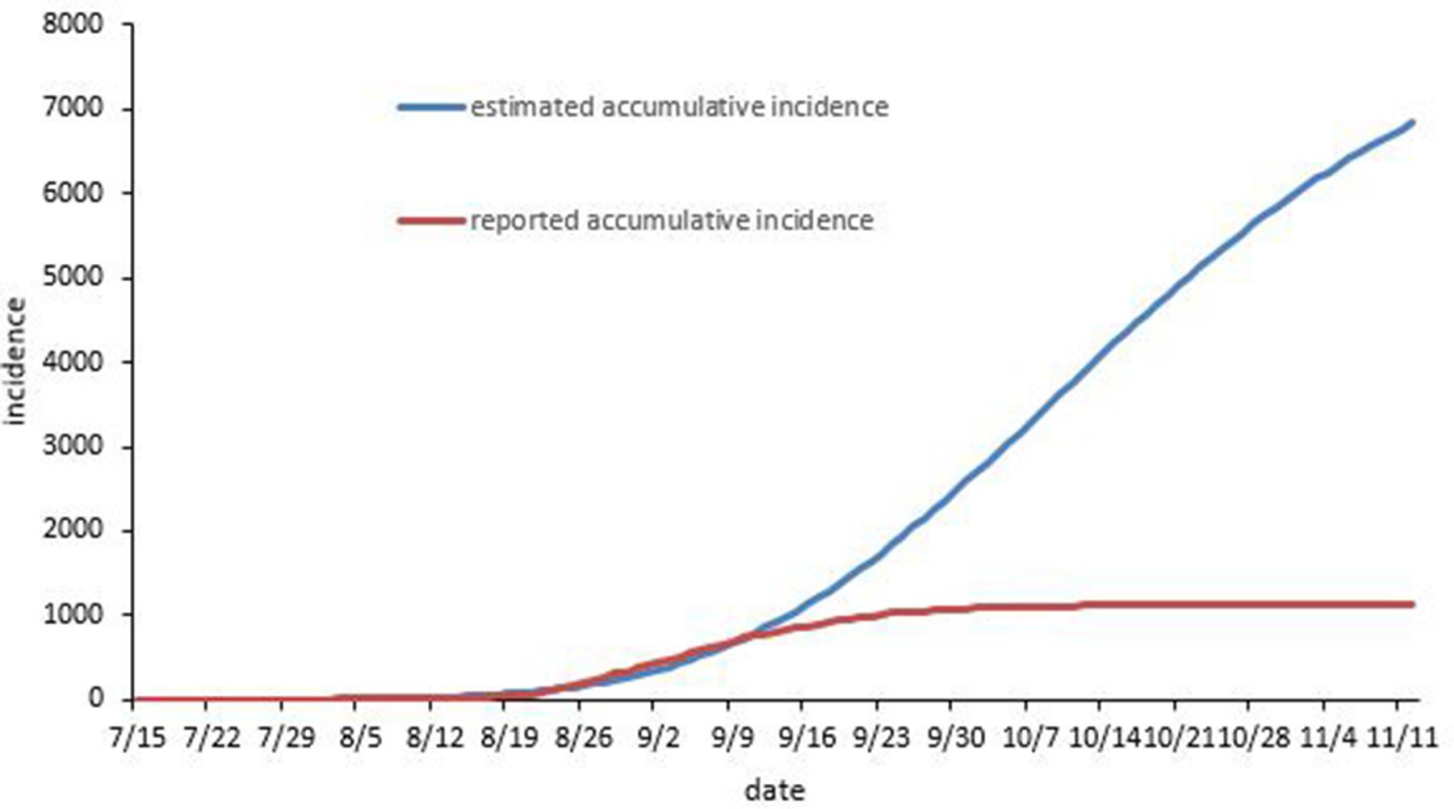
The temporal distribution of the accumulative reported cases and the simulated epidemics with no intervention.

After the unknown model parameters were estimated, the epidemic without any intervention was fitted by these parameters as shown in Table 1. Fig 3 shows that the estimated accumulative incidence was significantly higher than the reported accumulative incidence. The results show that the MAPE of the modeling performance was 30.72%. The minimum MAPE was 0.46%. The estimated value and the actual data were moderately matched (Fig 3).

**Fig 4:**
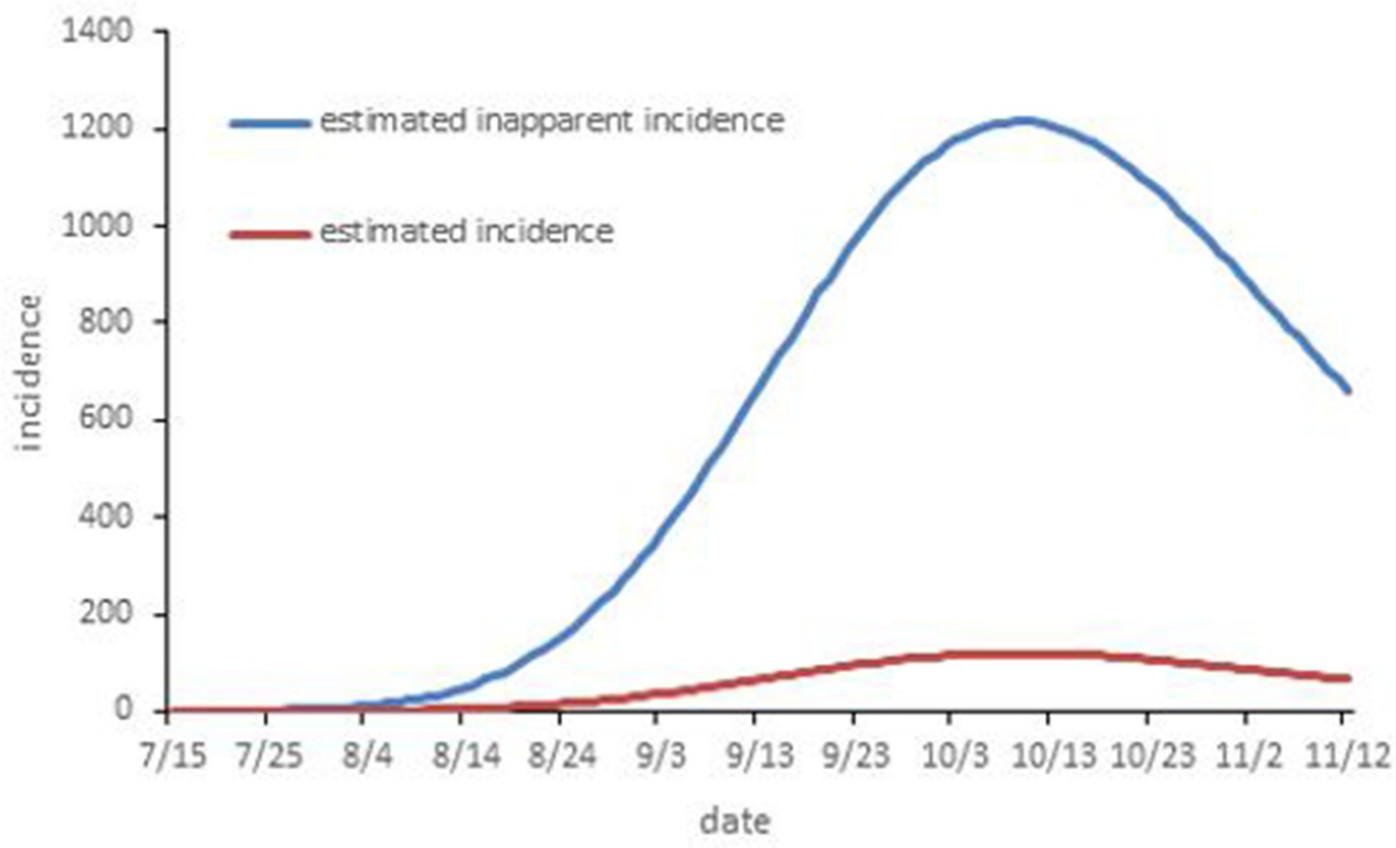
The temporal distribution of the estimated symptomatic case and the inapparent infections with no intervention.

According to the simulation without any intervention, there were 6090 indigenous dengue cases in total by the end of November 2, 2017. The peak epidemic occurred in 119 daily cases from October 8 to 11. The total reported cases were reduced by 81.33% in contrast to the estimated incidence without intervention. The inapparent incidence was also fitted at the same time. The inapparent cases increased quickly from the start of August and reached a peak of 1214 daily cases in October 9. There were a total of 62005 inapparent infections by the end of November 2. The *p* was 0.156, and the average daily inapparent cases were 10.18 times more than the symptomatic cases (Figs 3 and 4).

### A simulated epidemic with different levels of intervention measures

In the following three sections of the paper, the effectiveness of vector control with different patterns will be estimated. It is indicated that the reduction in the mosquito density could decrease the number of human infections and shorten the period of the epidemic (Fig 3A). Moreover, Fig 3A suggests that the higher the daily reduction in mosquito density, the lower the epidemic magnitude is. To illustrate, 1400 indigenous dengue cases were found at 5% daily reduction in mosquito density and 980 cases at 7.5%, and when daily reduction in mosquito density increased to 10%, the number of cases dropped to 773 by the end of November 2. The durations of these three epidemics were 114, 97 and 86 days, respectively. The peak of the epidemic also shifted to an earlier date on September 7, September 2, and August 31.

Next, we estimated the effectiveness of different initial times of implementing vector control, setting the daily reduction in mosquito density at 5%. Fig 3B indicates that earlier vector control could drastically reduce the number of human infections and shorten the epidemic period. By the end of November 2, indigenous dengue infections were 509, 1400, and 2279 for starting times of August 14, August 29, and September 8, respectively. The duration of the epidemic was 95, 114 and 125 days, respectively. The number of cases at different initial times, in contrast to the estimated incidence without intervention, was reduced by 91.64%, 77.01% and 62.58%, respectively.

**Fig 5:**
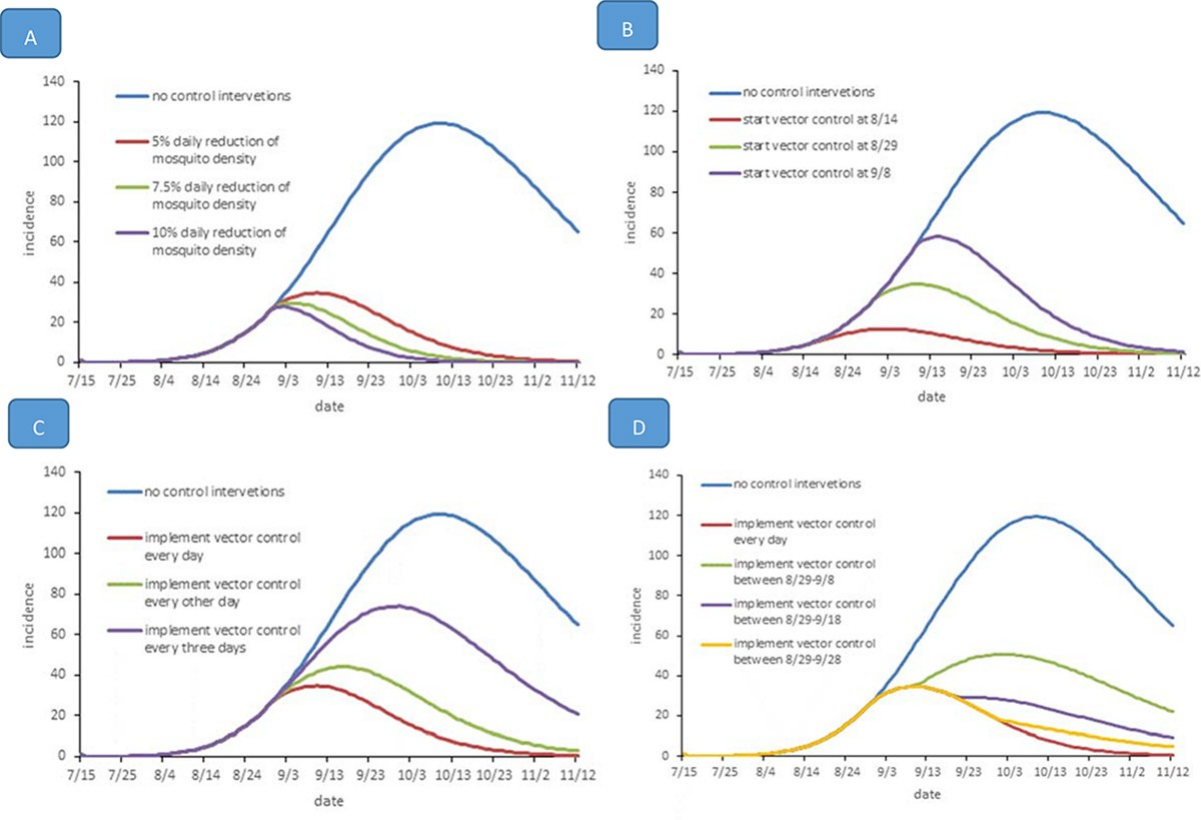
Effectiveness of different levels of vector control interventions on dengue outbreaks. **(A**) The effect of different levels of daily reduction in mosquito density. The starting time of the intervention was set at August 29. (**B**) The effect of different initial times of implementing vector control. The daily reduction in mosquito density was set to 5%. **(C**) The effect of different intervals of implementing vector control. The starting time of the intervention was set at August 29 and the daily reduction in mosquito density was set to 5%. (**D**) Comparisons of the effect of implementing the vector control for a continuous period. The starting time of the intervention was set at August 29, and the daily reduction in mosquito density was set to 5%.

A similar result was obtained for the impulse vector control measures with the daily reduction in mosquito density set to 5%. When implementing the vector control every day, every other day and every three days, 1400, 2050 and 3874 indigenous dengue cases were reported by the end of November 2 (Fig 3C). Surprisingly, even when vector control was implemented every three days, the magnitude of the epidemic would still decrease by 36.39% compared to the estimated incidence without intervention. Furthermore, the effectiveness of implementing vector control for ten days, twenty days and one month was estimated (Fig 3D). Interestingly, the effectiveness of implementing the vector control for a month is similar to that of implementing vector control every day. The total number of cases by the end of November 2 was 1574 with a month constant measure, which is slightly higher than the cases implementing the vector control every day (n = 1400). The indigenous dengue infections were 2937 and 1931 by the end of November 2 when vector control was employed for ten days and twenty days, respectively. It seems that the effectiveness of implementing the vector control with a 5% reduction in mosquito density for more than twenty days is satisfactory. The continuous vector control for more than twenty days is better than implementing the control measures every few days (Fig 5).

**Fig 6:**
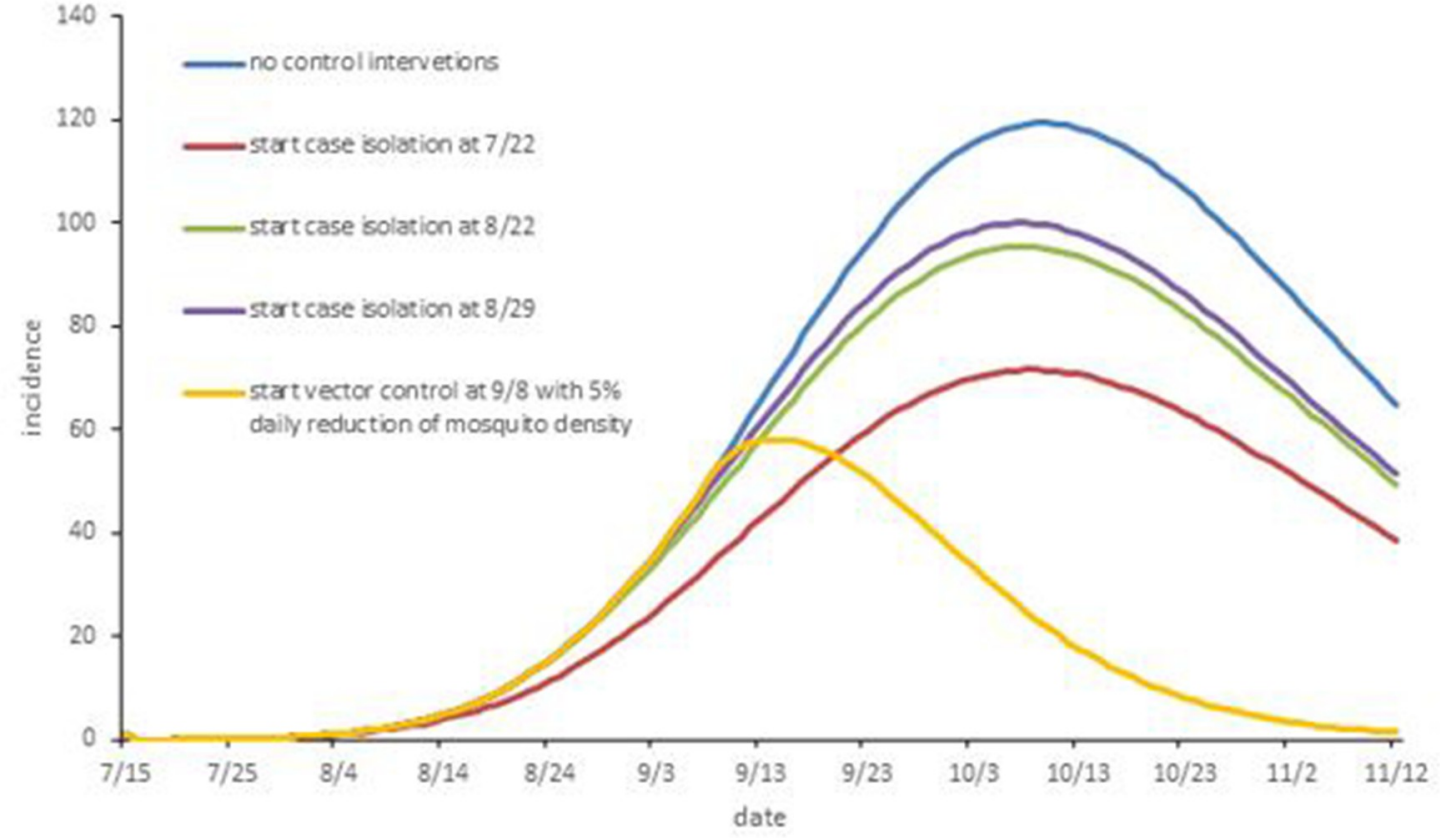
Comparison of the effect of different levels of case isolation and with the effect of implementing vector control.

Despite the fact that dengue is a mosquito-borne viral disease and the inapparent infection can also transmit it, we still estimated the effectiveness of isolation for symptomatic cases to examine whether this control measure is necessary. Fig 4 indicates that the case isolation was not satisfactory for controlling the development of the epidemic. There were a total of 3770, 5031 and 5261 indigenous dengue cases by the end of November 2 for starting time on July 22, August 22, and August 29, respectively. The epidemic was only reduced by 38.10% in contrast to the estimated incidence without intervention, even if case isolation began seven days after the onset of the first case. Better results are shown from the implementation of vector control for ten days (or starting at September 8 with a 5% daily reduction in mosquito) in comparison to that of isolating the symptomatic case (Fig 6).

### Sensitivity analysis

**Fig 7:**
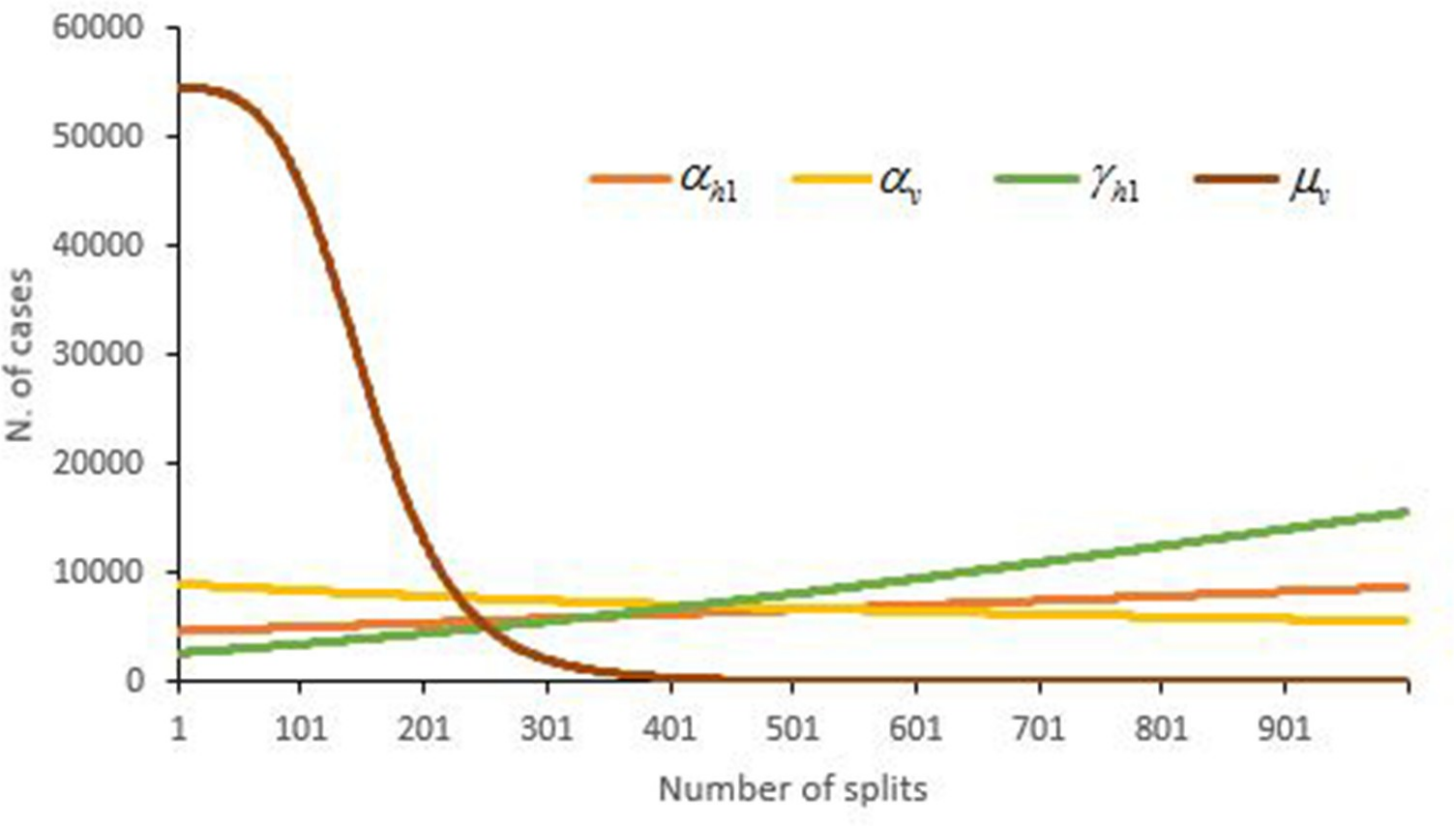
Sensitivity analysis of the parameters *α*_*h*1_, *α*_*v*_, *γ*_*h*1_ and *μ*_*v*_ of the outbreak in Hangzhou in 2017.

The total number of dengue cases by November 2 were very sensitive to the density-independent death rate for mosquitoes (*μ*_*v*_). However, the number of dengue cases was relative stable in relation to changes in the other three parameters, especially if the rate of humans changed from the exposed state to the symptomatic state and the rate of mosquitoes changed from the exposed state to the infectious state (Fig 7).

## Discussion

The dengue outbreak that occurred in Hangzhou was the most serious epidemic in the history of Zhejiang Province, China. The epidemic lasted nearly four months. The combination of rapid development of globalization, suitable meteorological conditions, high susceptibility of the population and delayed diagnosis of imported dengue cases led to this outbreak [8,38–40]. The gender and age distribution in this outbreak were attributed to the exposure chances [8].

Because the outbreak occurred in multiple areas at the county level, we evaluated the spatial correlation. According to the semivariogram, the semivariance was relatively low in the short range and gradually grew to the maximum. This means that the closer the counties are located geographically, the more similar the number of the dengue cases were and the stronger the regional impact the disease was. The ratio of the partial sill to the sill is the key indicator, which represents the extent of variation due to spatial autocorrelation. In the study, this ratio was 83.67%, which suggested that the spatial autocorrelation was relatively strong in general and that the variation due to the spatial autocorrelation was the main contribution to the observed difference. The result of global autocorrelation analysis also presented a similar conclusion. The cases affected each other across the counties by importing the disease from one area to another. For this reason, we considered all cases in different counties as one cluster.

One important finding is that the epidemic magnitude decreased significantly under the practical control intervention measures. This result is consistent with prior research [11,41] and provides evidence that the practical control measures in this outbreak are highly effective.

Another important finding is that the number of inapparent cases was far higher than the number of symptomatic infections. This high I:S ratio is almost the same as the research conducted in Huangpu town, China[28]. Furthermore, similar results were identified in previous studies of dengue outbreaks conducted in Cuba, Colombia, Singapore and Taiwan [42]. The inapparent rates were 78% to 94%. The inapparent rate was affected by many potential factors[42]. In our study, this high I:S ratio may be due to the type of vector and host immunity[28]. In this outbreak, only *Aedes albopictus* and *Culex* were identified, and only the *Ae*. *albopictus* pool was positive for dengue virus[8]. Moreover, *Aedes albopictus* is thought to transmit the virus at low titers, which is associated with low levels of viremia in patients and would result in less clinically overt or severe disease [28,43,44]. Another possible explanation is that since this is the first DF outbreak in Hangzhou on record, most of the patients were the primary infection who had poor immunity and resistance against the disease [8]. When compared to secondary infections, the I:S ratio for primary infection is significantly higher[18,45,46]. A similar discovery was found in a cohort study in Brazil [42]. Furthermore, human and viral genetics may play an important role in the outcome of infection, and some candidate genes were found to be related to the variation in the symptoms [42]. Nevertheless, the mechanism and the crucial element that affected the outcome of infection need to be determined more clearly in future studies. Overall, the inapparent cases play an important role in transmitting the disease during the outbreak that occurred in Hangzhou. Due to the large number and asymptomatic of the inapparent cases, new approaches to vector control are needed. One important measure is the adaptation of the endosymbiotic bacterium *Wolbachia* from *Drosophila* to *A*. *aegypti*, which has both life-shortening effects on the mosquito and direct transmission-blocking effects on dengue virus[2]. Another is the release of the genetically modified *A*. *aegypti* carrying a dominant lethal gene[2]. However, the effectiveness of these measures to control the *Aedes albopictus* should be tested.

We also found that high efficacy vector control measures can significantly decrease the number of cases, which is consistent with previous studies[11,47]. However, the intervention measures may be limited by the control resources. It is suggested that effective vector control measures should be implemented if there were adequate control resources. Otherwise, the vector control measures should be implemented at an earlier time. Similar to some studies[11,48], the number of cases would substantially decrease if vector control measures were started in a timely manner after the onset of the endemic epidemics. In our model, the number of cases would be reduced by more than 90%, even if the vector control measures were implemented one month after the onset of the first endemic case. As mentioned above, the delay of the diagnosis of the early cases is an important risk factor for controlling the spread of the epidemic in time. Therefore, the premise of implementing timely vector control is diagnosing and reporting cases at an early stage. It would be necessary to have a screening of the nonstructural1 (NS1) antigen in all suspected cases in all medical institutions among the season of the epidemic.

Furthermore, we found that continuous vector control intervention was necessary, which is consistent with previous research in this field[11]. The effect of implementing vector control measures every few days was not satisfactory compared to that of eradicating the mosquitoes every day. Our study also demonstrated that implementing vector control measures every day for 30 days could obtain similar effectiveness of performing the same work until the end of the epidemic. However, it should be noted that this time will be more than 30 days if the reduction in the mosquito density is less than 5%.

According to our study, isolation of the symptomatic case is not a good way to control the development of dengue epidemics due to the large number of inapparent infections. The effectiveness may be better when the I:S ratio is kept at a low level. It should be noted that the case management would use more resources and time. Furthermore, this measurement is actually difficult to implement when there is no enforcement rule to restrict the behavior of the case. Therefore, the actual effectiveness of isolation would be greatly reduced compared to the theoretical value.

The results of the sensitivity analysis indicated that the change in the value of the density-independent death rate for mosquitoes would affect the epidemic magnitude to a large extent. The possible reason was the wide range (8–42 days) of survival time of mosquitoes. The infected mosquitoes could transmit the virus to more people as its survival time is prolonged. Otherwise, there is less opportunity for mosquitoes to transmit the virus as its survival time shortens because the minimum time from the exposed state to the infectious state for mosquitoes requires at least 7 days.

Several limitations should be noted in our study. First, the estimated parameters were averaged in our model. In reality, the individual differences in humans and mosquitoes make the parameters different (e.g., transmission rate or recovery rate). The stochastic model may be used to provide a more accurate estimation for the epidemic in the future[11]. Second, there was no serological evidence to support the proportion of inapparent infections. Therefore, this proportion has to be estimated by the model and may lead to overestimation or underestimation of the epidemic. Third, the parameters for mosquitoes, such as the density-independent death rate, the transmission rate and the natural growth rate, are affected by many climate factors, especially temperature[49]. The daily mean temperature in Hangzhou is ordinarily below 20 degrees after October 15 and the density of the mosquitoes drops with temperature. Therefore, the effectiveness of the control measures in this epidemic may be overestimated. The model with climate factors is necessary for future studies and may provide more accurate simulations. Finally, owing to hypersensitivity to the number of cases for one parameter, the implications of our conclusion should be limited to the value setting in this research.

## Conclusion

In conclusion, the practical control interventions in the outbreak that occurred in Hangzhou were highly effective and prevented most of the infections. The proportion of inapparent infections was large, and inapparent infections played an important role in transmitting the disease during this epidemic. High efficacy and early vector control measures could significantly decrease the number of cases. Continuous vector control intervention was necessary compared with implementing them every few days. The effectiveness of isolation of the symptomatic case was not as good as that of controlling the mosquitoes for the high I:S ratio. As inapparent infections contribute to the global spread of dengue and increase the probability of severe outcomes in secondary infections, early, continuous and high efficacy vector control interventions are necessary to limit the development of dengue epidemics and to lower the future risk of severe outcomes. The timely diagnosis and case report are important in the intervention at an early stage of the epidemic.

## Supporting information

S1 File The database of key information on dengue infections in Hangzhou. (XLS)
